# Industrial Scale Isolation, Structural and Spectroscopic Characterization of Epiisopiloturine from *Pilocarpus microphyllus* Stapf Leaves: A Promising Alkaloid against Schistosomiasis

**DOI:** 10.1371/journal.pone.0066702

**Published:** 2013-06-26

**Authors:** Leiz M. C. Véras, Vanessa R. R. Cunha, Filipe C. D. A. Lima, Maria A. Guimarães, Marianne M. Vieira, Yuri D. M. Campelo, Vanessa Y. Sakai, David F. Lima, Paulo S. Carvalho Jr, Javier A. Ellena, Paulo R. P. Silva, Luciene C. Vasconcelos, Markus Godejohann, Helena M. Petrilli, Vera R. L. Constantino, Yvonne P. Mascarenhas, José Roberto de Souza de Almeida Leite

**Affiliations:** 1 Núcleo de Pesquisa em Biodiversidade e Biotecnologia, Universidade Federal do Piauí, Parnaíba, Piauí, Brazil; 2 Programa de Pós-Graduação em Biotecnologia – RENORBIO, Universidade Federal do Piauí, Teresina, Piauí, Brazil; 3 Departamento de Química Fundamental – Instituto de Química, Universidade de São Paulo, São Paulo, SP, Brazil; 4 Departamento de Física dos Materiais e Mecânica – Instituto de Física, Universidade de São Paulo, São Paulo, SP, Brazil; 5 Programa de Mestrado em Biotecnologia, Universidade Federal do Piauí, Parnaíba, Piauí, Brazil; 6 Departamento de Física e Informática – Instituto de Física de São Carlos, Universidade de São Paulo, São Carlos, SP, Brazil; 7 Anidro do Brasil Extrações S.A., Parnaíba, Piauí, Brazil; 8 Bruker BioSpin GmbH, Rheinstetten, Germany; Concordia University Wisconsin, United States of America

## Abstract

This paper presents an industrial scale process for extraction, purification, and isolation of epiisopiloturine (EPI) (2(3H)-Furanone,dihydro-3-(hydroxyphenylmethyl)-4-[(1-methyl-1H-imidazol-4-yl)methyl]-, [3S-[3a(R*),4b]]), which is an alkaloid from jaborandi leaves (*Pilocarpus microphyllus* Stapf). Additionally for the first time a set of structural and spectroscopic techniques were used to characterize this alkaloid. EPI has shown schistomicidal activity against adults and young forms, as well as the reduction of the egg laying adult worms and low toxicity to mammalian cells (*in vitro*). At first, the extraction of EPI was done with toluene and methylene chloride to obtain a solution that was alkalinized with ammonium carbonate. The remaining solution was treated in sequence by acidification, filtration and alkalinization. These industrial procedures are necessary in order to remove impurities and subsequent application of the high performance liquid chromatography (HPLC). The HPLC was employed also to remove other alkaloids, to obtain EPI purity higher than 98%. The viability of the method was confirmed through HPLC and electrospray mass spectrometry, that yielded a pseudo molecular ion of *m/z* equal to 287.1 Da. EPI structure was characterized by single crystal X-ray diffraction (XRD),^ 1^H and ^13^C nuclear magnetic resonance (NMR) in deuterated methanol/chloroform solution, vibrational spectroscopy and mass coupled thermal analyses. EPI molecule presents a parallel alignment of the benzene and the methyl imidazol ring separated by an interplanar spacing of 3.758 Å indicating a π-π bond interaction. The imidazole alkaloid melts at 225°C and decomposes above 230°C under air. EPI structure was used in theoretical Density Functional Theory calculations, considering the single crystal XRD data in order to simulate the NMR, infrared and Raman spectra of the molecule, and performs the signals attribution.

## Introduction

Alkaloids are organic compounds found in plant kingdom, fungus, bacteria and animals. The majority of these natural substance exhibits an alkaline character related particularly to the presence of basic amino groups (frequently heterocyclic) in their chemical structure. The word *alkaloid* is derived from the Latin “alkali” (basic) with the suffix “-oid” (-like). In the literature, many authors claim that a true alkaloid is composed by one or more basic nitrogen atoms in a heterocyclic ring, beside the intense biological activity in the presence of living organisms [1]. Additionally, alkaloids are known to be used as therapeutical agents for anesthetics, analgesics, and psycho-stimulant, among other pharmacological activities.

Over the past one hundred and fifty years, thousands of alkaloids have been isolated [2], and several high standard techniques can now be employed to evaluate the pharmacological and toxicological activities of these substances. Thus, new applications of centenarians alkaloids have been discovered, as the case of Pilocarpine, which has been isolated in 1875 and applied for decades in the treatment of glaucoma and, recently, also used for in the treatment of xerostomy [3], [4].

Several alkaloids have been isolated from the *Pilocarpus* genus, but many of them are still in analysis to evaluate their therapeutic applications [5]. The jaborandi species (*Pilocarpus* sp.) provides several molecules of the alkaloid class such as: pilocarpine, isopilocarpine, pilocarpidine, isopilocarpidine, pilosine, isopilosine, epiisopilosine, epiisopiloturine, 13–7-noria (11)-dehydro-pilocarpine, N,N-dimethyl-5-methoxy-tryptamine, N,N-dimethyl tryptamine, plastidesmine (1H)-4-methoxy-2-quinolone and dictamine [5].

The alkaloid epiisopiloturine (EPI) in particular was identified by Voightlander et al. in 1973 [6]. This substance has shown *in vitro* activity against *Schistosoma mansoni* [7], [8], the main agent of schistosomiasis, a neglected disease of poor, rural and forgotten populations. The parasitic disease represents one of the main public health problems in more than 70 tropical and subtropical countries, especially in Africa, Asia and Latin America[9]. Approximately 240 million of people have been infected worldwide and about 700 million of people are at risk areas [10], [11].

In adults and young (schistosomula) forms of *S. mansoni*, the EPI alkaloid had schistomicidal activity at a concentration of 150 µg/mL and 300 µg/mL, respectively [7], [9]. Besides the death, the sub lethal dose (100 µg/mL) of this alkaloid promotes the total reduction of the egg laying in the paired adult form, differently from what has been observed when praziquantel (the drug reference standard) was used in the treatment of schistosomiasis. Furthermore, the EPI was not cytotoxic in the peritoneal macrophages, and can be obtained, in large scales, by sustainable and economical process [7], [8].

The activity against *S. mansoni*, no toxicity to mammalian cells and the plant extraction by sustainable form turn up the studies and characterization of EPI indispensable. The meticulous investigation of the waste from the extraction of a natural product could introduce an important strategy for the discovery and development of a new drug [7].

This work reports the industrial extraction, purification and isolation of EPI from jaborandi leaves, and also its physicochemical characterization by single crystal X-ray diffraction, ^1^H and ^13^C nuclear magnetic resonance (NMR) in deuterated methanol/chloroform solution, vibrational infrared (FT-IR) and Raman (FT-Raman) spectroscopies, and mass coupled thermal analyses. The spectroscopic characterization data are further supported by computational calculations performed in the framework of the Density Functional Theory (DFT).

## Materials and Methods

### Industrial extraction, pre-purification and isolation of alkaloid from jaborandi leaves

**Figure 1:**
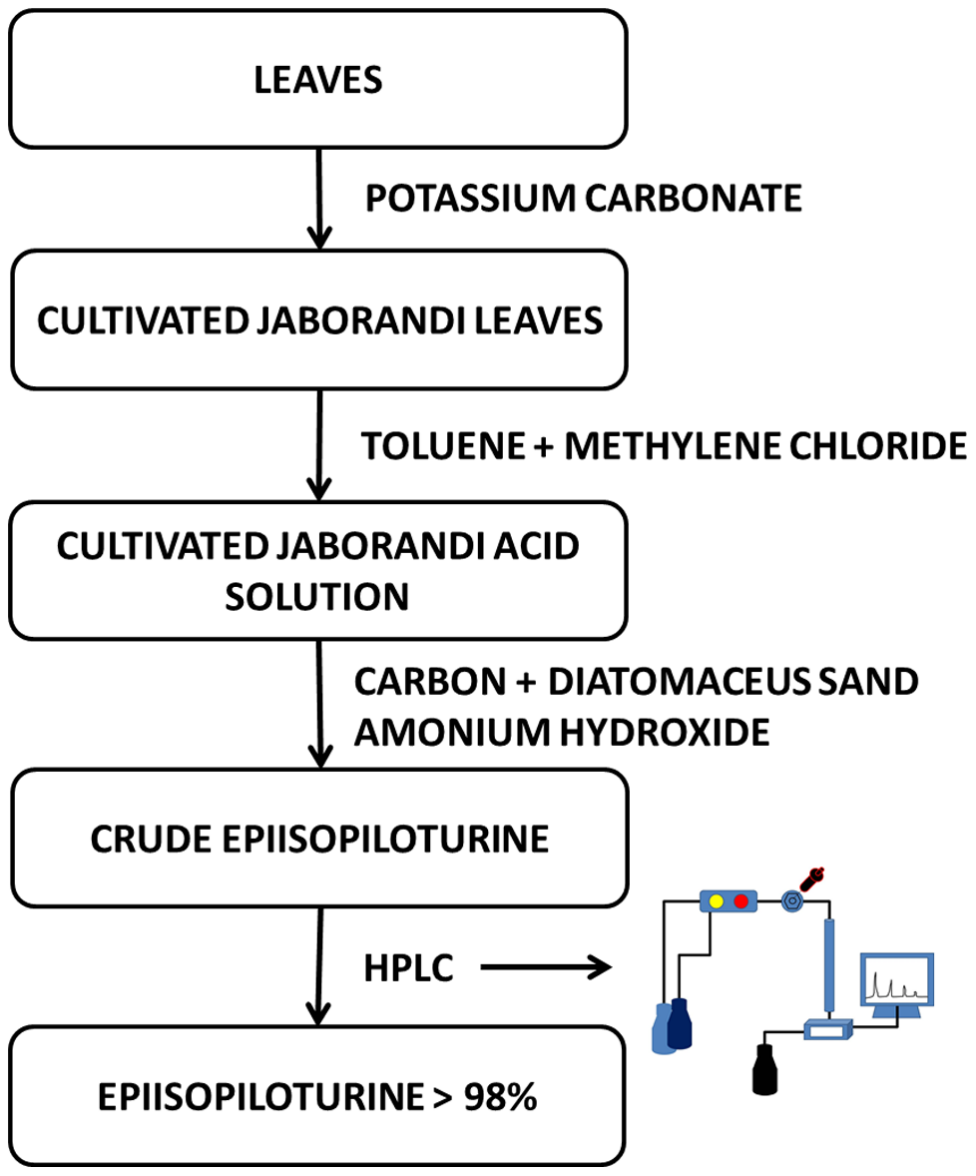
Scheme of all necessary steps in obtaining Epiisopiloturine with >98% purity from Jaborandi leaves.

The initial step is based on the alkalinization of 1,500 kg of jaborandi leaves with a solution of potassium carbonate, followed by the extraction of all alkaloids (solid-liquid extraction) with toluene and methylene chloride solvents (**Figure 1**). The organic phase was submitted to liquid-liquid extraction with an aqueous solution of sulfuric acid. Hereafter, 250 L of the aqueous solution with a mean content of 2% (m/v) of EPI in the sulfate salt form was cooled, alkalinized with ammonium hydroxide solution and treated with activated carbon and diatomaceous sand.

After treatment with carbon and diatomaceous sand, the impure EPI was dissolved in an aqueous solution containing hydrochloric acid and filtered on a pressure lentil filter (filter medium: cloth/two filter papers/cloth) under reduced pressure. The filtrate containing EPI hydrochloride was alkalinized with ammonium hydroxide solution to precipitate the EPI neutral form, and then the solution was filtered under reduced pressure [8]. The aim of the previous step was to remove the impurities such as carbon and diatomaceous sand in order to submit EPI to further purification process by high performance liquid chromatography (**Figure 1**) (all details from this steps is in patently process – 000121-INPI, Brazil).

The crude *EPI* (**Figure 1**) at a concentration of 10 mg/mL was dissolved in the mobile phase with potassium phosphate 5% (v/v), filtered by a membrane of 0.45 µm and set to the preparative high performance liquid chromatography – HPLC (SHIMADZU Prominence, AUTOSAMPLER SIL-10AF, CTO-20A, DGU-20A5, LC-6AD, CBM-20A, SPD-20A, Tokyo, Japan).

The preparative chromatographic conditions set were performed in a column of LiChrospher 60 RP Select B (250×25 mm, 5 µm). The mobile phase was 367.59 mM potassium phosphate adjusted at pH 2.5 with a flow rate of 10 mL/min for a time run of 90 min. The detection was done using a UV detector at a wavelength of 216 nm and the column oven was set to 50°C.

The injection volume was 1000 µL and 500 mL fractions were collected with a concentration of 100 mg/L of crude EPI. The solution obtained after preparative HPLC was alkalinized between pH 9 to 9.5 and subjected to liquid-liquid extraction with industrial chloroform. The organic phase was evaporated with a vacuum controller V-850, a water bath – B – 491 and an evaporator – R – 215 (BUCHI, Switzerland).

For fine analyses an analytical HPLC was employed to verify each process step, with the LiChrospher 60 RP Select B (250×4.6 mm, 5 µm), using external standard (EPI standard at 20 µg/mL and pilocarpine standard at 50 µg/mL, Merck, Darmstadt, Germany). The mobile phase was 367.59 mM potassium phosphate adjusted at pH 2.5. The flow rate was 1 mL/min, the column oven was set to 50°C for a time run of 50 min with UV detection at a wavelength of 216 nm.

The molecular mass confirmation was performed by mass spectrometry (AmaZon SL, Bruker Daltonics, Bremen – Germany), which was used in positive electrospray ionization mode. The capillary voltage was – 1,800 V, temperature at 250°C, and the mass spectra were acquired in mass range of *m/z* 160 – 300 Da. MS/MS was carried out in manual mode with fragmentation of the precursor ion by collision induced dissociation (CID) using helium (He) as the collision gas. Precursor ions were selected within an isolation width of 2 u and scans were accumulated with variable RF signal amplitudes. The *m/z* scale of the mass spectrum was calibrated using the external calibration standard G2421A electrospray ‘*tuning mix*’ from Agilent Technologies (Santa Rosa, USA).

### Physical measurements

#### Nuclear Magnetic Resonance (NMR)

10 mg of EPI was dissolved in 2 mL of CD_3_OD/CDCl_3_ 1∶1 mixture. Afterward 600 µL of this solution was transferred to a 5 mm NMR sample tube. For the measurements of NMR data, standard parameter sets created for the Bruker CMC-se (complete molecular confidence-structure elucidation) program were uniformly employed. Gradient COSY (Correlation Spectroscopy) (2 scans per increment) and ^1^H -^13^C HMBC (Heteronuclear Multiple-Bond Correlation spectroscopy) (8 scans per increment) were acquired using 4 k complex data points in F2 and 512 points in F1 dimension. A 1H-15N HMBC was acquired with 32 scans per increment with a time domain of 256 in F1 and 2 k points in F2. The multiplicity edited gradient HSQC (Heteronuclear Single Quantum Correlation) (2 scans per increment) was acquired with 2k data points in F2 and 400 points in the F1 dimension. The instrument used was an AVANCE III 600 MHz NMR spectrometer equipped with a 5 mm TXI probe head (Bruker Biospin, Rheinstetten, Germany). The NMR data acquired were processed according to the general experimental procedures.

The Bruker structure elucidation package CMC-se (Topspin 3.1) was used to get the peak and multiplet lists in a fully automated way. The multiplet lists are collected into a correlation table. The automatic step is usually followed by short visual inspection of the results. The SELU module contains combined display linking the correlation table and the spectra display. This allows fast inspection and correction of generated HSQC, HMBC and COSY multiplet lists. After the correlation table is populated and inspected, the structure generator calculates structures which are consistent with the NMR data acquired. Finally an independent ^13^C chemical shift prediction method is used to validate the result.

#### Single crystal X-ray diffraction (XRD)

The X-ray diffraction data were collected at room temperature using a KAPPA-CCD Diffratometer with MoKα radiation (λ = 0.71073 Å). The cell refinements were performed using the software Collect and Scalepack [12]. The final unit cell parameters were obtained on all reflections. Data reduction was carried out using the software Denzo-SMN and Scalepack. The structure was solved by Direct Methods and anisotropically refined with full-matrix least-squares on F^2^ using SHELXL97 [13]. The hydrogen atoms bonded to C and N atoms were positioned geometrically and refined with riding constraints with distance restraints of N-H = 0.86 Å, aromatic C-H = 0.93 Å and with U_iso_(H) = 1.2 U_eq_(N,C). The crystallographic data were deposited at the Cambridge Crystallographic Data Center under the numbers CCDC 915132. Copies of the data can be obtained, free of charge, via www.ccdc.cam.ac.uk.

#### Vibrational FT-IR and FT- Raman

FT-IR spectrum of EPI sample diluted in KBr was recorded in the 4000–400 cm^−1^ range on a Bomen spectrophotometer, model MB-102, with a coupled diffuse reflectance accessory (Pike Technologies, Inc.). FT-Raman spectrum was recorded in a FT-Raman Bruker FRS-100/S spectrometer using 1064 nm exciting radiation (Nd:YAG laser Coherent Compass 1064–500 N) and a Ge detector.

#### Mass coupled thermal analyses (TGA-DSC-MS)

The thermal analyses were recorded on a Netzsch thermoanalyser model TGA/DSC 490 PC Luxx coupled to an Aëolos 403 C mass spectrometer, using a heating rate of 10°°C/min and under synthetic air flow of 50 mL/min.

### Computational analysis

The Density Functional Theory (DFT) [14] in the Kohn-Sham (KS) scheme [15] was used to investigate the electronic structure, vibrational properties and NMR ^13^C and ^1^H isotropic chemical shifts for the isolated EPI alkaloid molecule. The B3LYP [16] exchange correlation functional and the 6–311++G** basis set were used as implemented in the Gaussian 09 computational package [17]. Gauge Including Atomic Orbitals (GIAO) method [18]–[20] and diffuse functions in the basis set were applied for the calculation of the C and H NMR spectra. All simulations were carried out in vacuum conditions and T = 0 K.

## Results and Discussion

**Figure 2:**
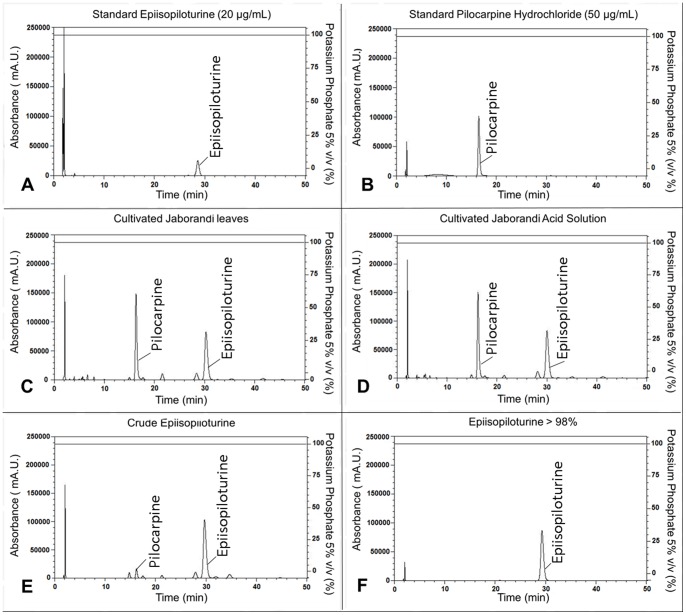
Analytical HPLC used LiChrospher 60 RP column and eluted with potassium phosphate . (A) Standard EPI (20 µg/mL), (B) Standard pilocarpine (50 µg/mL), (C) “cultivated jaborandi leaves” solution, resulted from first extraction step, (D) “cultivated jaborandi acid” solution, obtained EPI under salt form, (E) Solution of “crude EPI” with some impurities as pilocarpine and other alkaloids, (F) last step of isolation showing EPI >98% purity.

**Figure 3:**
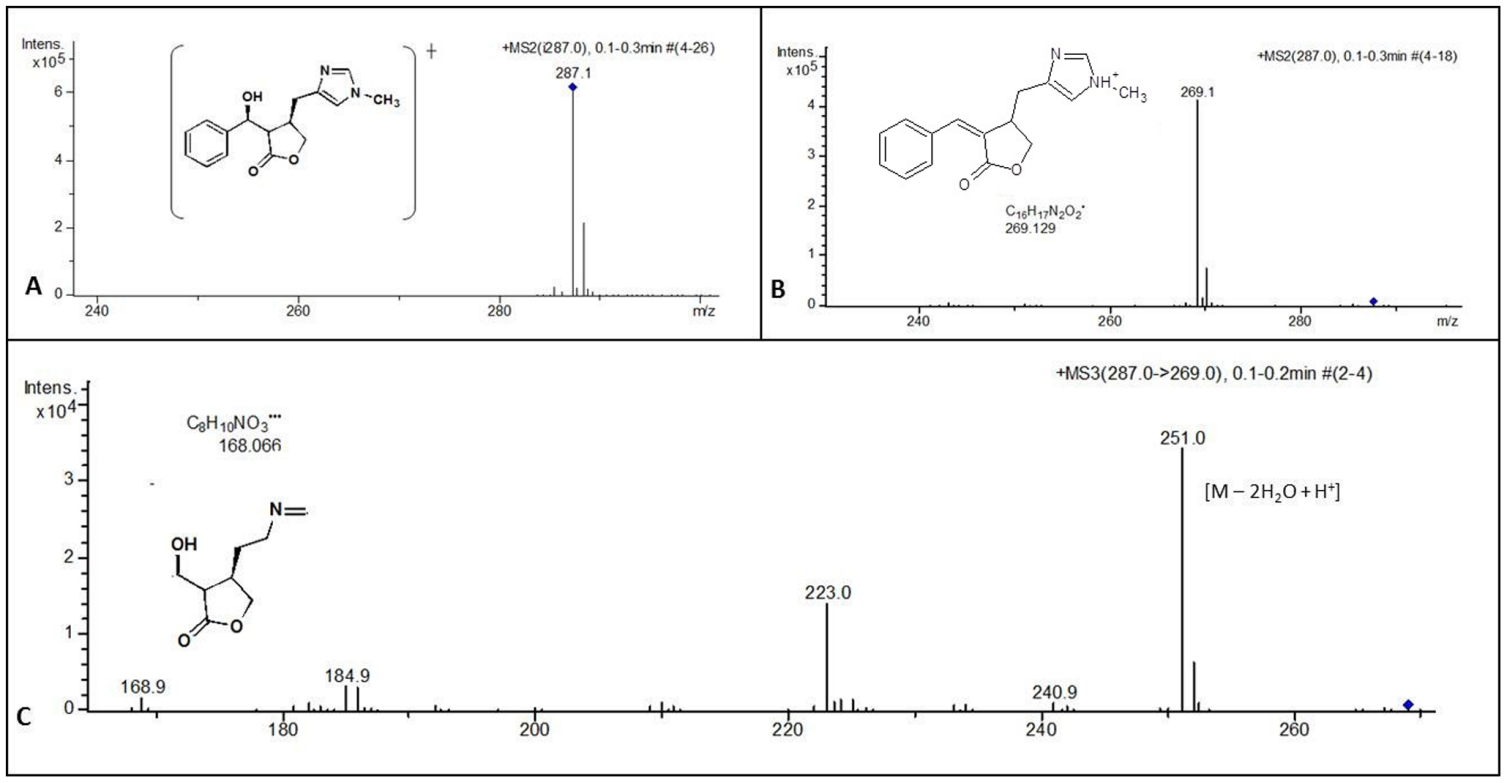
Mass spectrum obtained from ESI+/Ion Trap. (A) free EPI with a pseudo molecular ion m/z 287.1 Da [M+H]^+^, (B) MS^2^ with characteristic fragment at m/z 269.1 Da [M – H_2_O + H]^+^, (C) MS^3^ with fragments at m/z 251.0 Da [M – 2H_2_O + H^+^] and 168.06 Da with proposed chemical structure.

The results, here presented, describe a methodology for extraction, purification and isolation of EPI from jaborandi leaves. The HPLC (**Figure 2**) and LC/MS ESI+/Ion Trap (**Figure 3**) techniques have demonstrated the purity and initial characterization of each process step. For industrial scale, 1,500 kg of jaborandi leaves have been used through all steps described in **Figure 1**, what lead to obtain around 2 kg of pure EPI, used in the structural and spectroscopic characterization described in this work.

The MS/MS analysis showed a pseudomolecular ion with *m/z* 287.1 Da [M + H]^+^ and a MS^2^ fragment with *m/z* 269.1 Da [M – H_2_O + H]^+^. This “molecular fingerprint” of EPI has been previously reported in the literature[6]. Structural and spectroscopic characterizations were also performed to assure the integrity of the EPI molecule.

**Figure 4:**
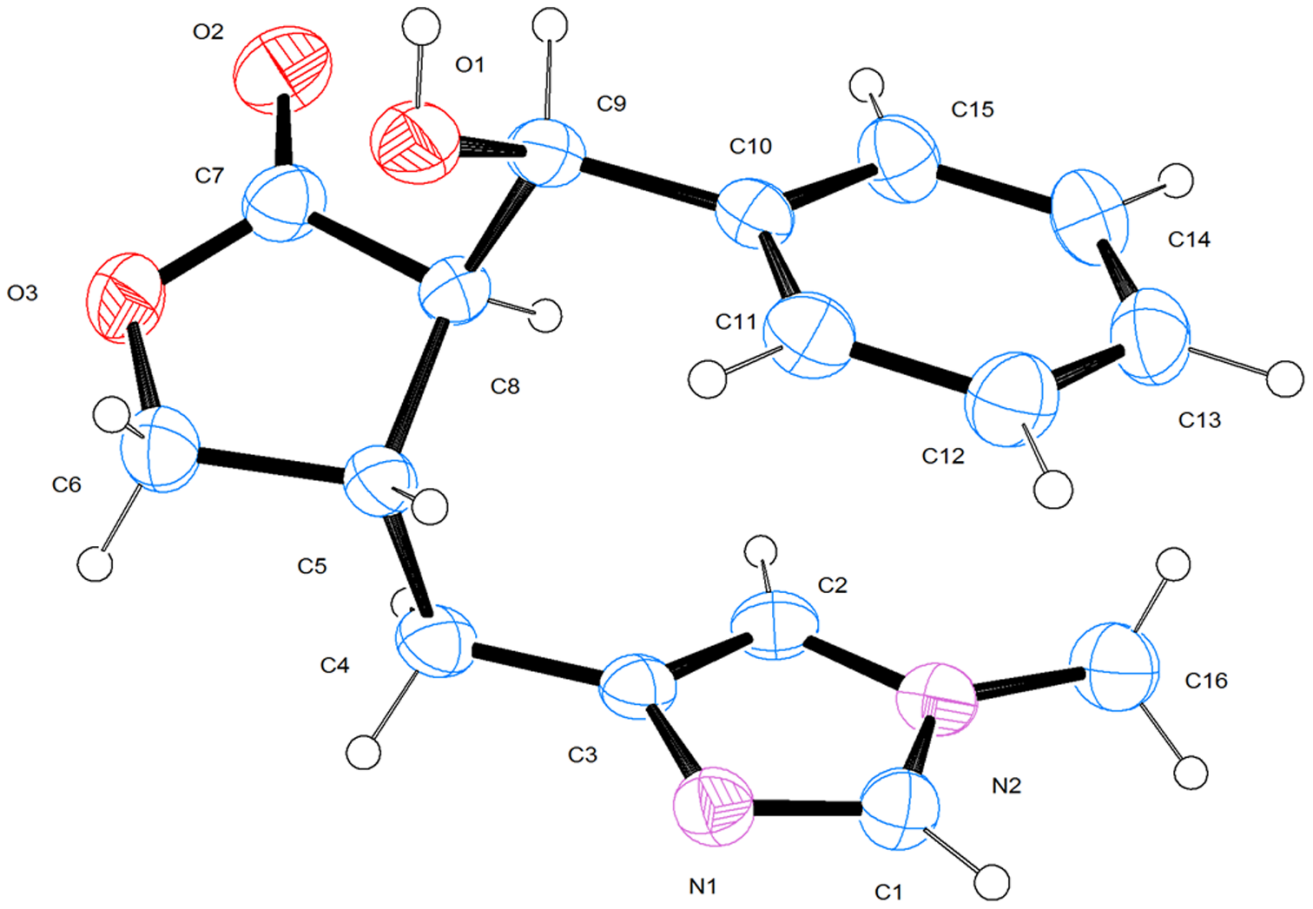
Isolated Epiisopiloturine molecular structure.

**Figure 5:**
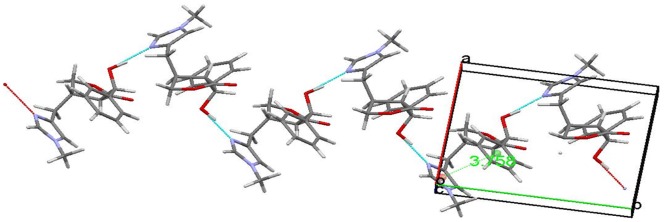
Epiisopiloturine Crystalline form with the molecules represented in stick format. Color code: carbon (gray), hydrogen (white), nitrogen (blue) and oxygen (red). Cyan lines are only guide lines to illustrate hydrogen bonds between the hydroxyl group and the imidazole ring of neighboring molecules in the solid.

The EPI molecular structure is shown in **Figure 4**, created by the ORTEP [21] software. An interesting feature of this molecule is the parallel alignment of the benzene and the methyl imidazole ring separated by an interplanar spacing of 3.758 Å, indicating a π-π bond interaction. Another feature of the crystalline packing is the presence of a hydrogen bond between hydroxyl H group, from one molecule, and the N1 of the methyl imidazole ring of another, forming in this way a continuous chain of hydrogen bonded molecules, as can be seen in **Figure 5**.

**Table 1** shows the differences between experimental X-ray diffraction and structural relaxation results obtained through DFT calculations, where we can notice that the largest absolute differences are related with hydrogen atoms. It can be seen, from the bond angle results shown in **Table 2**, that the maximum distortion difference is 5.64° in the C3-C2-H2 angle.

**Table 1:** EPI bond distances obtained through x-ray diffraction (Experimental) and DFT results (Calculated).

	Experimental (Å)	Calculated (Å)	Difference (Experimental -Calculated) (Å)
O1–C9	1.419 (3)	1.429	−0.010
O1–H1	0.82	0.963	−0.143
C1–N1	1.314 (3)	1.312	0.002
C1–N2	1.341 (3)	1.364	−0.023
C1–H1A	0.93	1.080	−0.150
N1–C3	1.379 (3)	1.379	0.000
C3–C2	1.359 (3)	1.374	−0.015
C3–C4	1.481 (3)	1.496	−0.015
N2–C2	1.369 (3)	1.381	−0.012
N2–C16	1.452 (3)	1.453	−0.001
–	1.511 (3)	1.520	−0.009
C9–C8	1.536 (3)	1.543	−0.007
C9–H9	0.98	1.098	−0.118
C5–C4	1.531 (3)	1.546	−0.015
C5–C6	1.532 (3)	1.539	−0.007
C5–C8	1.537 (3)	1.539	−0.002
C5–H5	0.98	1.090	−0.110
C2–H2	0.93	1.078	−0.148
C10–C11	1.383 (3)	1.396	−0.013
C10–C15	1.390 (3)	1.398	−0.008
C4–H4A	0.97	1.095	−0.125
C4–H4B	0.970	1.095	−0.125
C8–C7	1.515 (3)	1.533	−0.018
C8–H8	0.980	1.092	−0.112
O3–C7	1.345 (3)	1.353	−0.008
O3–C6	1.446 (3)	1.448	−0.002
C16–H16A	0.960	1.092	−0.132
C16–H16B	0.960	1.089	−0.129
C16–H16C	0.960	1.092	−0.132
O2–C7	1.207 (3)	1.199	0.008
C11–C12	1.381 (3)	1.394	−0.013
C11–H11	0.930	1.083	−0.153
C15–C14	1.382 (3)	1.392	−0.010
C15–H15	0.930	1.086	−0.156
C6–H6A	0.970	1.090	−0.120
C6–H6B	0.970	1.090	−0.120
C13–C12	1.371 (4)	1.392	−0.021
C13–C14	1.376 (4)	1.394	−0.018
C13–H13	0.930	1.084	−0.154
C12–H12	0.930	1.084	−0.154
C14–H14	0.930	1.085	−0.155

Atom labels accordingly to Figure 4.

**Table 2:** EPI bond angles obtained through x-ray diffraction (Experimental) and DFT results (Calculated).

	Experimental (°)	Calculated (°)	Difference(Experimental – Calculated) (°)
C9–O1–H1	109.50	108.21	1.29
N1–C1–N2	112.37 (19)	112.32	0.05
N1–C1–H1A	123.80	125.88	−2.08
N2–C1–H1A	123.80	121.80	2.00
C1–N1–C3	105.62 (17)	105.54	0.08
C2–C3–N1	108.60 (18)	109.82	−1.22
C2–C3–C4	130.68 (19)	129.22	1.46
N1–C3–C4	120.60 (18)	120.95	−0.35
C1–N2–C2	106.14 (17)	106.27	−0.13
C1–N2–C16	126.60 (20)	126.76	−0.16
C2–N2–C16	127.24 (19)	126.96	0.28
O1–C9–C10	112.88 (16)	112.61	0.27
O1–C9–C8	107.18 (16)	106.45	0.73
C10–C9–C8	111.68 (16)	113.75	−2.07
O1–C9–H9	108.30	109.79	−1.49
C10–C9–H9	108.30	108.25	0.05
C7–C8–C5	103.81 (17)	103.80	0.01
C9–C8–C5	114.85 (17)	116.96	−2.11
C7–C8–H8	109.20	107.96	1.24
C9–C8–H8	109.20	107.18	2.02
C5–C8–H8	109.20	111.91	−2.71
C7–O3–C6	109.86 (17)	110.48	−0.62
N2–C16–H16A	109.50	110.89	−1.39
N2–C16–H16B	109.50	110.70	−1.2
H16A–C16–H16B	109.50	108.64	0.86
N2–C16–H16C	109.50	108.87	0.63
H16A–C16–H16C	109.50	109.10	0.4
H16B–C16–H16C	109.50	108.57	0.93
O2–C7–O3	121.60 (20)	122.96	−1.36
O2–C7–C8	126.90 (20)	127.01	−0.11
O3–C7–C8	111.48 (18)	110.03	1.45
C12–C11–C10	121.60 (20)	120.53	1.07
C8–C9–H9	108.30	105.75	2.55
C4–C5–C6	113.10 (17)	111.44	1.66
C4–C5–C8	112.45 (18)	112.38	0.07
C6–C5–C8	102.70 (17)	101.97	0.73
C4–C5–H5	109.50	108.19	1.31
C6–C5–H5	109.50	110.75	−1.25
C8–C5–H5	109.50	112.07	−2.57
C3–C2–N2	107.27 (17)	106.04	1.23
C3–C2–H2	126.40	132.04	−5.64
N2–C2–H2	126.40	121.80	4.6
C11–C10–C15	117.60 (20)	118.84	−1.24
C11–C10–C9	122.62 (18)	121.49	1.13
C15–C10–C9	119.79 (18)	119.67	0.12
C3–C4–C5	112.38 (16)	113.42	−1.04
C3–C4–H4A	109.10	108.57	0.53
C5–C4–H4A	109.10	109.84	−0.74
C3–C4–H4B	109.10	109.96	−0.86
C5–C4–H4B	109.10	108.00	1.10
H4A–C4–H4B	107.90	106.80	1.10
C7–C8–C9	110.25 (17)	108.68	1.57
C12–C11–H11	119.20	120.25	−1.05
C10–C11–H11	119.20	119.22	−0.02
C14–C15–C10	121.0 (2)	120.05	0.95
C14–C15–H15	119.50	120.09	−0.59
C10–C15–H15	119.50	119.85	−0.35
O3–C6–C5	107.00 (18)	106.26	0.74
O3–C6–H6A	110.30	107.92	2.38
C5–C6–H6A	110.30	111.82	−1.52
O3–C6–H6B	110.30	107.06	3.24
C5–C6–H6B	110.30	113.48	−3.18
H6A–C6–H6B	108.60	109.98	−1.38
C12–C13–C14	119.80 (20)	119.60	0.2
C12–C13–H13	120.10	120.22	−0.12
C14–C13–H13	120.10	120.18	−0.08
C13–C12–C11	119.9 (2)	120.26	−0.36
C13–C12–H12	120.00	120.09	−0.09
C11–C12–H12	120.00	119.65	0.35
C13–C14–C15	120.10 (20)	120.05	0.05
C13–C14–H14	120.00	120.09	−0.09
C15–C14–H14	120.00	119.85	0.15

Atom labels accordingly to Figure 4.

The EPI was further characterized through ^1^H and ^13^C NMR spectroscopy. The standard way of chemical shift assignments [22] was compared with theoretical DFT calculations for the isolated molecule and can be seen in **Supporting Information S1**. The ^1^H NMR data obtained in this work are in conformity with seminal results reported by Voigtlander et al [6].

**Figure 6:**
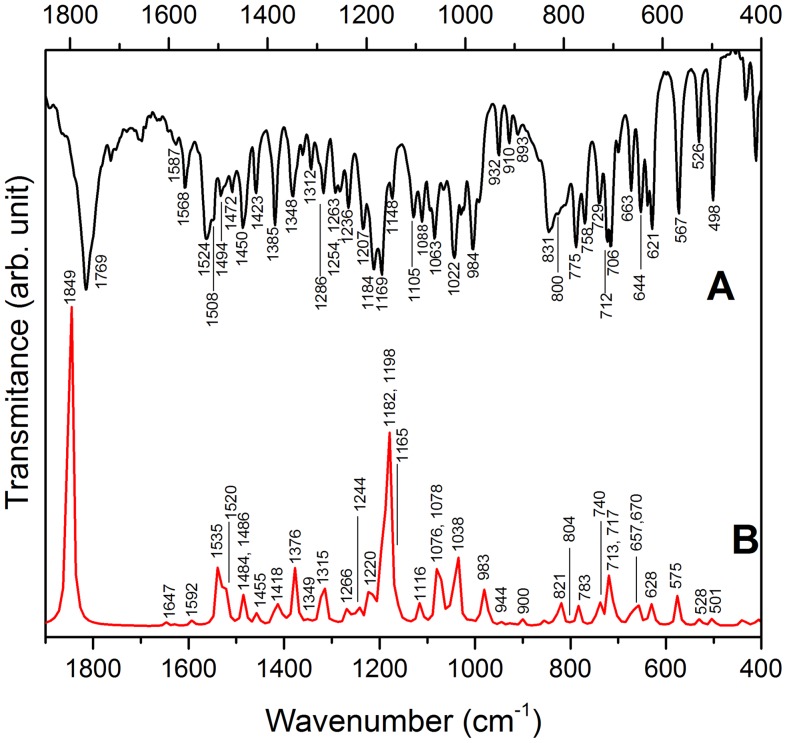
Epiisopiloturine FT-IR spectra: A) experimental and B) calculated.

**Figure 7:**
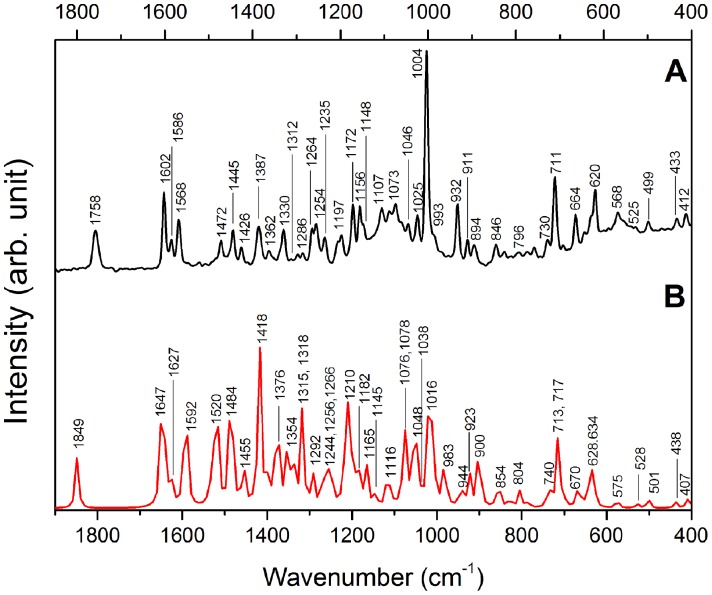
Epiisopiloturine FT-Raman spectra: A) experimental and B) calculated.

Measured and calculated FT-IR and FT-Raman EPI molecule spectra are shown in **Figure 6** and **Figure 7**. The vibrational band assignments, presented in **Table 3**, were proposed based on general literature about organic molecules [22]–[25] and a study reported about an isomer of EPI named epiisopilosine [26]. The vibrational assignments were supported by DFT calculations performed in this work as can be seen in **Table 3**. The very strong band at 1769 cm^−1^ is assigned to C = O stretching of the lactone ring, in the FT-IR spectrum shown in **Figure 6**, and corresponds to the medium intensity band at 1758 cm^−1^ in the FT-Raman spectrum, shown in **Figure 7**. The FT-Raman spectrum reveals three strong bands of EPI around 1602–1568 cm^−1^ (**Figure 7**) characteristic of the C-C stretching vibrational mode of benzene and imidazole rings; these bands are weak in the FT-IR spectrum. On the other hand, the bands around 1524–1494 cm^−1^, attributed to modes related to imidazole and lactone groups (see **Table 3**) are present in the FT-IR spectrum but practically absent in the FT-Raman spectrum. Well-defined band at 1385 cm^−1^ assigned to the scissoring C9-O1-H vibrational mode of the secondary alcohol group attached to the organic molecule is observed in both spectra. The strong band at 1004 cm^−1^ (FT-Raman) is attributed to the C-C-C trigonal bending of benzene, while the bands around 990–930 cm^−1^ are assigned to the bending of the lactone ring, in both FT-IR and FT-Raman spectra. Several bands attributed to the vibration of C-H group of benzene and imidazole rings can be observed in the 900–730 cm^−1^ range, as listed in **Table 3**. The low frequency region is dominated by bands associated to vibrations involving C-C-C of benzene, imidazole (600–500 cm^−1^) and also to all the EPI structure bending modes (430–400 cm^−1^).

**Table 3:** Infrared (IR) and Raman wavenumbers (cm^−1^) of solid state EPI.

*Epiisopiloturine*			*Assignment*
Experimental		Calculated	
IR	Raman		
	412	407	δ (all structure)
	433	438	δ (all structure)
498	499	501	C-C-C out of plane bending (benzene)
526	525	528	C-C-C out of plane bending (benzene)
567	568	575	C-C-C in-phase bending (benzene)
621	620	628	ring puckering (imidazole)
		634	C-C-C in-phase bending (benzene)
644		657	sc (O3-C7-C8)
663	664	670	C-C-C in-phase bending (benzene)
712	711	713	C-C-C in-phase puckering (benzene)
729		717	C-H out-of-plane in-phase (benzene)
758	730	740	C-H out of plane bend (imidazole)
775		783	C-H out-of-plane in-phase (benzene)
800 sh	796	804	ring puckering (imidazole)
831		821	C-H out of plane bend (imidazole)
	846	854	r CH_2_ (C4)
893	894	900	r CH_2_ (C4), r CH_2_ (C6)
910	911	923	C-H out of plane (benzene), r CH_2_ (C4)
932	932	944	r CH_2_ (C4), β (lactone)
984	993 sh	983	β (lactone)
	1004	1016	C-C-C trigonal bending
1022	1025	1038	β (lactone), r CH_2_ (C4)
	1046	1048	C-H in plane bending (benzene)
1063	1073	1076	w CH_3_ (C16)
1088		1078	w CH_3_ (C16), C-H in plane bending (benzene)
1105	1107	1116	t CH_2_ (C4), γ (lactone)
1148	1148	1145	w CH_3_ (C16)
1169	1156	1165	t CH_2_ (C4), γ (lactone)
1184	1172	1182	β (lactone)
1207		1198	C-H in plane bending (benzene), t CH_2_ (C4)
	1197	1210	C-H in plane bending (benzene)
1236		1220	t CH_2_ (C4), t CH_2_ (C6)
1254	1235	1244	β (lactone)
	1254	1256	t CH_2_ (C6), C-H in plane bending (imidazole)
1263	1264	1266	t CH_2_ (C4), t CH_2_ (C6), C-H in plane bending (imidazole)
1286	1286	1292	w CH_2_ (C4)
1312	1312	1315	C-C stretching (benzene)
	1330	1318	C-N stretching (imidazole) w CH_2_ (C4)
1348		1349	C-C stretching (benzene)
	1362	1354	w CH_2_ (C4)
1385	1387	1376	sc(C9-O1-H), νas(C8-C9-C10)
1423	1426	1418	νs(N1-C1-N2), w CH_3_ (C16), νas(C16-N2-C1)
1450	1445	1455	w CH_3_ (C16)
1472	1472	1484	C-C stretching (benzene)
1494		1486	sc CH_3_ (C16)
1508		1520	sc CH_2_ (lactone), w CH_3_
1524		1535	νas N-C-N (imidazole)
1568	1568	1592	ν C-C (imidazole)
1587	1586	1627	ν C-C (benzene)
	1602	1647	ν C-C (benzene)
1769	1758	1849	ν(C = O lactone)

Calculated vibrational wavenumbers (cm-1) for the isolated EPI molecule. A tentative assignment of the observed vibrational modes is also shown. See text for theoretical details. ν =  stretching, δ =  bending, β =  bending in plane, γ =  bending out of plane, r =  rocking, τ =  twist, sc =  scissoring, ω =  wagging, νs =  symmetric stretching, νa =  antisymmetric stretching, sh  =  shoulder.

**Figure 8:**
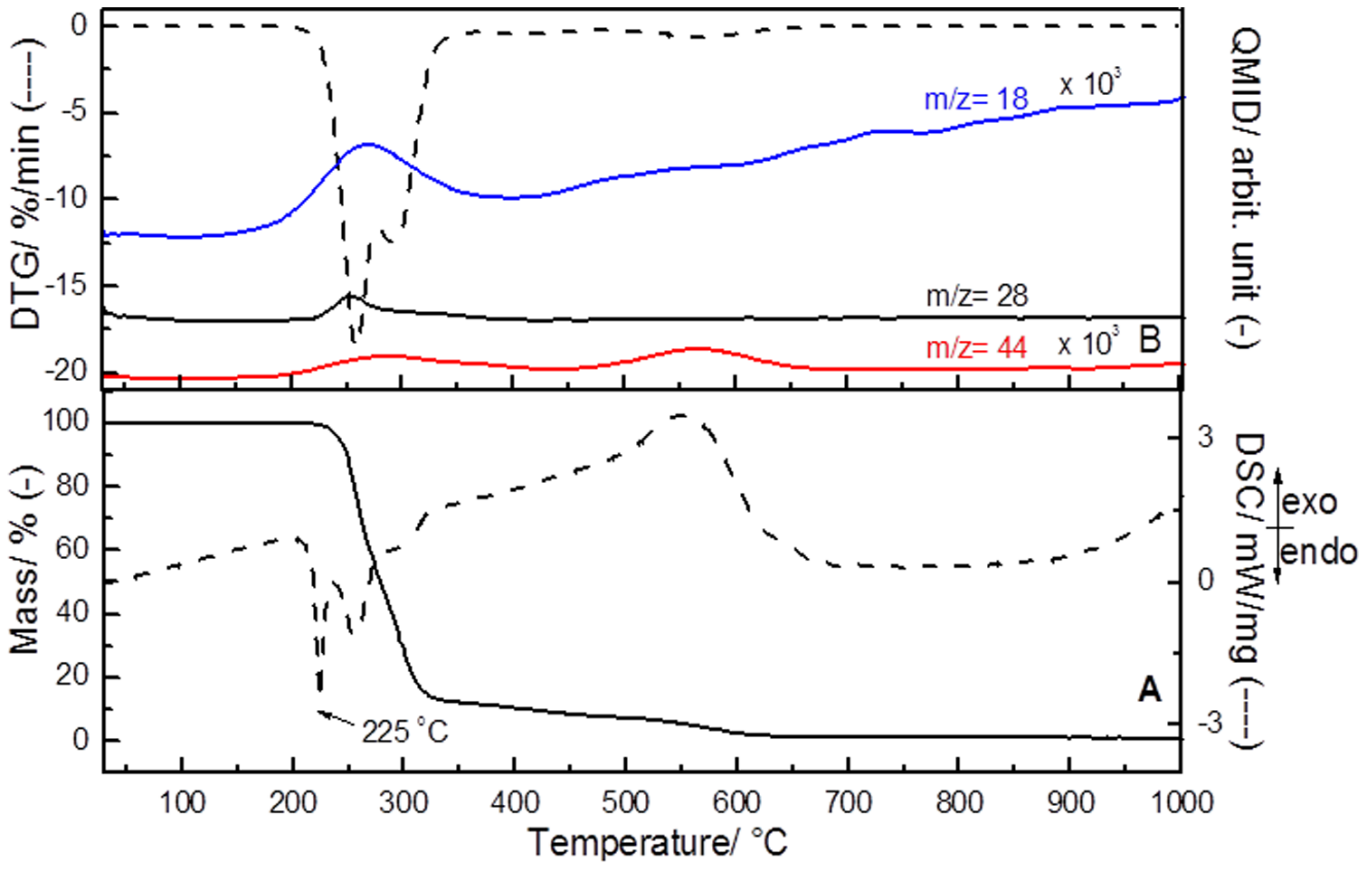
Epiisopiloturine TGA-DSC (A) and DTG-MS (B) curves under air atmosphere.

The TGA-DSC-MS curves of the isolated EPI are shown in **Figure 8**, where three events can be observed. The first event (an endothermic process) occurs at 225°C, and can be attributed to the melting process of the imidazole alkaloid (**Figure 8A**). Partly according to Voigtlander et al [6], the melting point of EPI is about 218–219°C when measured using a copper block. In line with the DTG-MS curves (**Figure 8B**), EPI is decomposed around 230–350°C (ca. 87 wt. %), in air atmosphere, producing water (*m/z*  = 18), carbon monoxide (*m/z*  = 28) and carbon dioxide (*m/z*  = 44) molecules. The third event observed in the temperature range between 360–695°C (12 wt.%) is related to the decomposition of the remaining organic molecule, producing H_2_O and CO_2_. Under the experimental conditions used in this work, no other released gases were identified. A residual product of 0.5 wt.% can be attributed to some impurities from the raw material and/or introduced during the process of EPI isolation.

## Conclusion

This work describes, for the first time, an industrial process to obtain EPI in high purity. The treatment with low-polarity solvents combined with HPLC technique allowed the isolation of the EPI alkaloid from jaborandi leaves. The technique of ESI/Ion Trap allowed attesting that its purity is higher than 98%, besides fragment characteristics of imidazole alkaloids produced by MS^n^. Single crystal X-ray diffraction data has shown the structure of the EPI molecule as well as its arrangement in solid state. The ^1^H and ^13^C NMR, IR and Raman spectroscopy data were supported by DFT simulations. Each assay had their contribution to characterize the EPI, allowing the interpretation of the experimental data which shows the integrity of the molecule isolated by the procedures of extraction and purification presented in this paper. According to TGA-DSC data, EPI melts at 225°C, and undergoes decomposition mainly in the 230–350°C range under air atmosphere.

The results presented in this work contribute to the advance of the isolation of EPI and provide a set of structural, spectroscopic and thermal properties of the alkaloid molecule. This study supports efforts to develop EPI as a new antiparasitic agent.

## Supporting Information

Figure S1 **Experimental (black) and theoretical (red) ^1^H NMR EPI spectra.**
(TIF)

Figure S2 **Experimental (black) and theoretical (red) ^13^C NMR EPI spectra.**
(TIF)

Supporting Information S1 **Chemical Shift Assignments.**
(DOCX)

Table S1 **EPI ^1^H and ^13^C NMR chemical shifts.** Atom labels accordingly to Figure S1.
(DOC)
