# Supplementary figures and images for: Industrial Scale Isolation, Structural and Spectroscopic Characterization of Epiisopiloturine from Pilocarpus microphyllus Stapf Leaves: A Promising Alkaloid against Schistosomiasis
Source: PLoS One. 2013 Jun 26;8(6):e66702. doi: 10.1371/journal.pone.0066702 (PMC3694155; doi:10.1371/journal.pone.0066702)

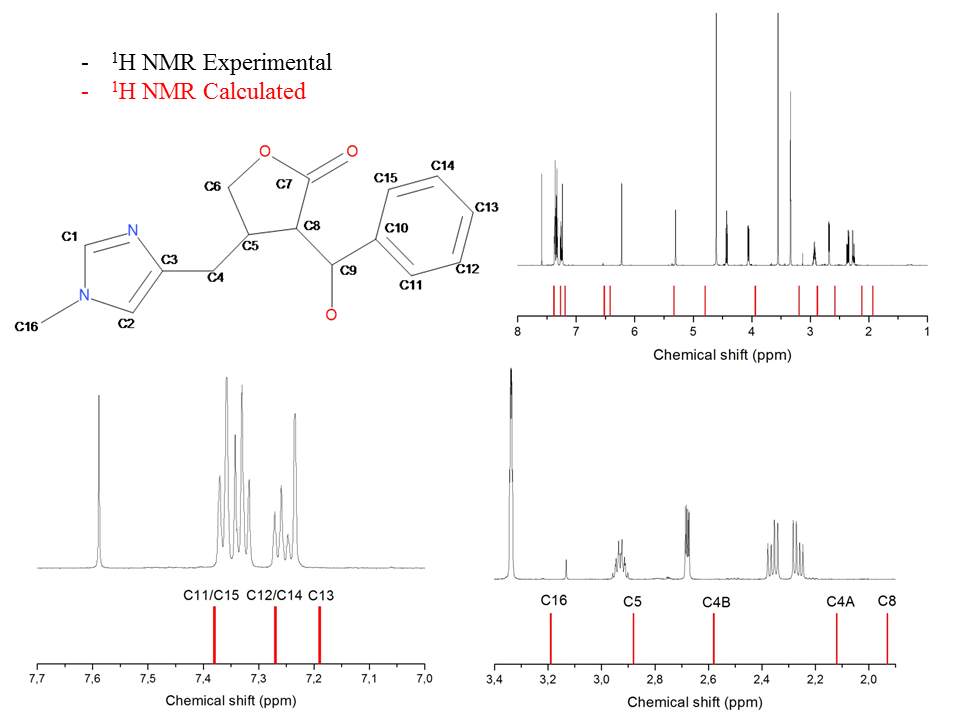

Supplement: Figure S1 — Experimental (black) and theoretical (red) 1H NMR EPI spectra. (TIF) [file pone.0066702.s001.tif]

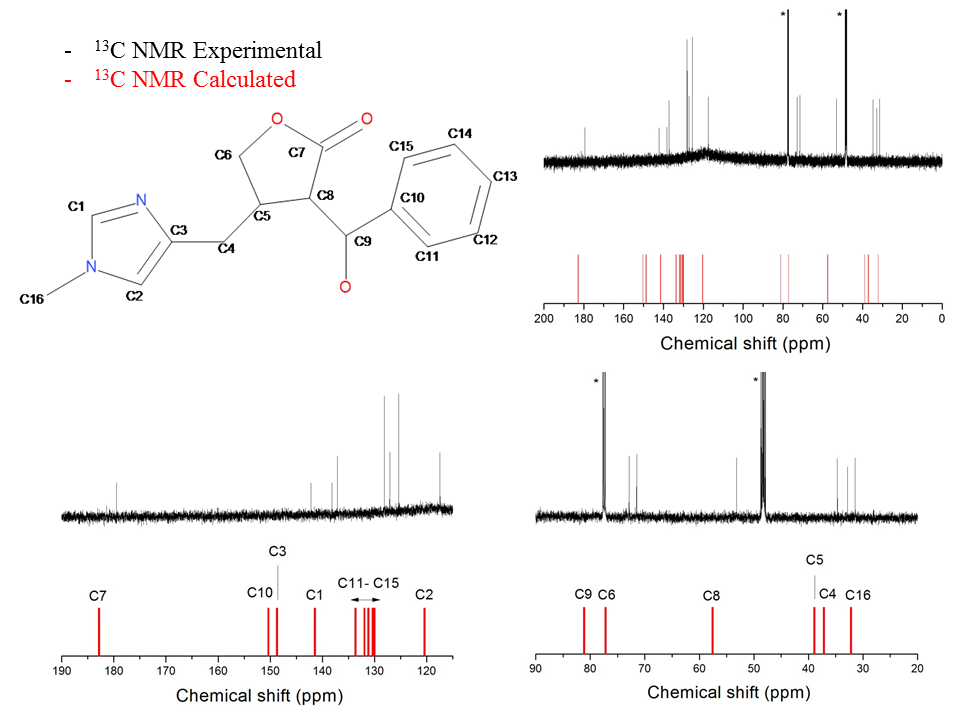

Supplement: Figure S2 — Experimental (black) and theoretical (red) 13C NMR EPI spectra. (TIF) [file pone.0066702.s002.tif]
