# Supplementary material for: Industrial Scale Isolation, Structural and Spectroscopic Characterization of Epiisopiloturine from Pilocarpus microphyllus Stapf Leaves: A Promising Alkaloid against Schistosomiasis
Source: PLoS One. 2013 Jun 26;8(6):e66702. doi: 10.1371/journal.pone.0066702 (PMC3694155; doi:10.1371/journal.pone.0066702)
Supplement: Supporting Information S1 — Chemical Shift Assignments. (DOCX) [file pone.0066702.s003.docx]

***Supporting Information***

***Chemical Shift Assignments***

The ^1^H and ^13^C NMR EPI spectra as well as the theoretical NMR spectra for the isolated molecule, obtained using DFT calculations, are shown in **Figure S1** and **Figure S2**. The standard way of chemical shift assignments[1] and the corresponding DFT calculations are shown in **Table S1**.Overall good agreement between the reported experimental data and the DFT calculations can be observed, what is a further indication of the integrity of the molecule after the extraction and isolation processes as well as the reliability of the model compound. The ^1^H NMR spectrum of the EPI shows the presence of the H from the CH_3_ group (C16) at 3 ppm. It is observed the difference between the H (C9) at 5.30 ppm and the H (C4) at around 2.3 ppm due to the deshielding of the H from the hydroxyl group. Moreover, the H from the imidazole and benzene aromatic ring at 7 ppm is deshielded by the resonance. The H (C6 and C7) is slightly more deshielded than the H (C5 and C8) due to the lactone group. The observed NMR signals for hydrogen atoms at C1, C2-C3 and C16 are in agreement with the Voigtlander et al. work[2].

The ^13^C NMR spectrum of the EPI presents the peaks related to the CH_3_ and CH_2_ groups (C16 and C4) at 32-31 ppm and CH (C9) at 71 ppm, where the latter is deshielded by the hydroxyl group compared to the C4, likewise was observed for the ^1^H NMR spectrum. The C1 and C3 from the imidazole ring are deshielded due to the resonance of the aromatic ring. Additionally, the resonance peaks at 130-133 ppm related to the CH (C11, C12, C13, C14 and C15) of the benzene ring are slightly less deshielded than the C1 and C3 from the imidazole aromatic ring. Finally, downfield is observed for the C7, from the lactone ring, that is deshielded due to the linkage to the oxygen atom, whereas the CH_2_ (C6) directly joined to the C-O (lactone group) and C8 (CH) are shielded by the ring.

The inversion of the calculated and experimental values seen in C4/C16 and C6/C9 (shown in **Table S1**) should be mainly due to the different conditions of the simulation compared to the experimental measurements. On one hand, the experimental spectrum was recorded dissolving EPI in MeOD solvent. On the other hand, the simulation has been carried out considering the molecule in vacuum conditions. C9 and C6 atoms are bonded to oxygen atoms that could be affected by the solvent through the establishment of H/D bonds. The C4 and C16 atoms should not be affected by the solvent as the groups mentioned above. It is also important to notice that EPI conformation in MeOD can be different from that one adopted in the solid state (spectrum simulation considered the molecule conformation obtained by XRD single crystal data). Nevertheless, the 1ppm deviations, in these cases, are in the experimental uncertainly.

***Supporting information references***

1. Silverstein R, Webster F, Kiemle D (2005) Spectrometric Identification of Organic Compounds. In: Brennan D, editor. New York: Wiley. pp. 204–244.

2. Voigtländer H-W, Balsam G, Engelhardt M, Pohl L (1978) Epiisopiloturin, ein neues Pilocarpus-Alkaloid. Archiv der Pharmazie 311: 927–935.
