# Supplementary material for: Industrial Scale Isolation, Structural and Spectroscopic Characterization of Epiisopiloturine from Pilocarpus microphyllus Stapf Leaves: A Promising Alkaloid against Schistosomiasis
Source: PLoS One. 2013 Jun 26;8(6):e66702. doi: 10.1371/journal.pone.0066702 (PMC3694155; doi:10.1371/journal.pone.0066702)
Supplement: Table S1 — EPI 1H and 13C NMR chemical shifts. Atom labels accordingly to Figure S1. (DOC) [file pone.0066702.s004.doc]

Table S1. EPI ^1^H and ^13^C NMR chemical shifts. Atom labels accordingly to Figure S1.

|  | **^1^H Shift (ppm)** | | **^13^C Shift (ppm)** | | |
| --- | --- | --- | --- | --- | --- |
| **Atom** | **Experimental** | **Calculated^a^** | **Atom** | **Experimental** | **Calculated** |
| **C4A** | 2.26 | 2.12 | C4 | 31.43 | 37.14 |
| **C4B** | 2.36 | 2.58 | C16 | 32.85 | 32.17 |
| **C8** | 2.68 | 1.93 | C5 | 34.69 | 38.90 |
| **C5** | 2.93 | 2.88 | C8 | 53.17 | 57.56 |
| **C16** | 3.55 | 3.19 | C9 | 71.49 | 81.13 |
| **C6A** | 4.05 | 3.94 | C6 | 72.88 | 77.20 |
| **C6B** | 4.43 | 4.80 | C2 | 117.43 | 120.40 |
| **C9** | 5.30 | 5.33 | C15/C11 | 125.36 | 130.18 |
| **C2** | 6.22 | 6.42 | C13 | 127.08 | 131.19 |
| **C1** | 7.23 | 6.52 | C12/C14 | 128.11 | 132.81 |
| **C13** | 7.26 | 7.19 | C1 | 137.12 | 141.43 |
| **C12/C14** | 7.33 | 7.27 | C3 | 138.18 | 148.72 |
| **C11/C15** | 7.36 | 7.38 | C10 | 142.20 | 150.37 |
|  |  |  | C7 | 179.47 | 182.84 |

^a^ DFT calculations described in the text.
